# The thiol-reductase activity of YUCCA6 enhances nickel heavy metal stress tolerance in Arabidopsis

**DOI:** 10.3389/fpls.2022.1007542

**Published:** 2022-09-27

**Authors:** Joon-Yung Cha, Song Yi Jeong, Gyeongik Ahn, Gyeong-Im Shin, Myung Geun Ji, Sang Cheol Lee, Dhruba Khakurel, Donah Mary Macoy, Yong Bok Lee, Min Gab Kim, Sang Yeol Lee, Dae-Jin Yun, Woe-Yeon Kim

**Affiliations:** ^1^ Research Institute of Life Sciences, Institute of Agriculture and Life Science, Gyeongsang National University, Jinju, South Korea; ^2^ Division of Applied Life Science (BK21four), Plant Molecular Biology and Biotechnology Research Center, Gyeongsang National University, Jinju, South Korea; ^3^ Department of Biology, Graduate School of Gyeongsang National University, Jinju, South Korea; ^4^ College of Pharmacy and Research Institute of Pharmaceutical Science, Gyeongsang National University, Jinju, South Korea; ^5^ Department of Biomedical Science and Engineering, Konkuk University, Seoul, South Korea

**Keywords:** auxin, heavy metal tolerance, nickel stress, reactive oxygen species, thiol-reductase

## Abstract

Anthropogenic activities cause the leaching of heavy metals into groundwater and their accumulation in soil. Excess levels of heavy metals cause toxicity in plants, inducing the production of reactive oxygen species (ROS) and possible death caused by the resulting oxidative stress. Heavy metal stresses repress auxin biosynthesis and transport, inhibiting plant growth. Here, we investigated whether nickel (Ni) heavy metal toxicity is reduced by exogenous auxin application and whether Ni stress tolerance in *Arabidopsis thaliana* is mediated by the bifunctional enzyme YUCCA6 (YUC6), which functions as an auxin biosynthetic enzyme and a thiol-reductase (TR). We found that an application of up to 1 µM exogenous indole-3-acetic acid (IAA) reduces Ni stress toxicity. *yuc6-1D*, a dominant mutant of *YUC6* with high auxin levels, was more tolerant of Ni stress than wild-type (WT) plants, despite absorbing significantly more Ni. Treatments of WT plants with YUCASIN, a specific inhibitor of YUC-mediated auxin biosynthesis, increased Ni toxicity; however *yuc6-1D* was not affected by YUCASIN and remained tolerant of Ni stress. This suggests that rather than the elevated IAA levels in *yuc6-1D*, the TR activity of YUC6 might be critical for Ni stress tolerance. The loss of TR activity in YUC6 caused by the point-mutation of Cys85 abolished the YUC6-mediated Ni stress tolerance. We also found that the Ni stress–induced ROS accumulation was inhibited in *yuc6-1D* plants, which consequently also showed reduced oxidative damage. An enzymatic assay and transcriptional analysis revealed that the peroxidase activity and transcription of *PEROXIREDOXIN Q* were enhanced by Ni stress to a greater level in *yuc6-1D* than in the WT. These findings imply that despite the need to maintain endogenous IAA levels for basal Ni stress tolerance, the TR activity of YUC6, not the elevated IAA levels, plays the predominant role inNi stress tolerance by lowering Ni-induced oxidative stress.

## Introduction

Heavy metals, such as nickel (Ni^2+^), cadmium (Cd^2+^), copper (Cu^2+^), zinc (Zn^2+^), cobalt (Co^2+^), chromium (Cr^2+^), mercury (Hg^2+^), lead (Pb^2+^), and arsenic (As^2+^), are naturally found in the Earth’s crust, but can be major soil pollutants. Their concentrations are increasing in many regions through a combination of natural processes and anthropogenic activities, including mining, agricultural practices, and industrialization (Briffa et al., 2020; Raffa et al., 2021). The release of heavy metals into habitats, particularly groundwater and soil, has become a great concern in agriculture due to their adverse effects on the growth and quality of crops and food safety (Rai et al., 2019). Heavy metal pollution is estimated to have an economic impact of more than US $10 billion per year worldwide, and in fact, it has been reported that fruit production in the contaminated area is reduced by 85.2% (Galal et al., 2021; Montaño-López and Biswas, 2021). Because all heavy metals are non-biodegradable, the remediation of heavy metal–contaminated soils is necessary to avoid their leaching and mobilization (Liu et al., 2018).

Plants absorb metals through the root systems and use some of them as micronutrients in a trace amount (Rengel, 1999); however, excess amounts of heavy metals cause metal toxicity, resulting in adverse effects on the molecular, biochemical, and physiological activities of plants (Tiwari and Lata, 2018).

Ni is an essential micronutrient for plants in trace amounts (0.01–10 µg Ni per g dry weight of plants) (Marschner, 2011); however, the excess application of fertilizers, animal manure, and pesticides, as well as other anthropogenic activities, have increased the release of Ni into the environment, resulting in Ni toxicity for plants (Chang et al., 1984). Their hyper-accumulation of Ni causes oxidative stress, disturbs their water balance, and suppresses photosynthesis in plant cells, which manifests as leaf necrosis and stunted growth of both the root and shoot (Ghori et al., 2019). As a non-redox metal, Ni generates reactive oxygen species (ROS), including various oxygen free radicals and hydrogen peroxide (H_2_O_2_), by indirect mechanisms that stimulate the ROS-producing enzyme NADPH oxidase, displacing essential cations from the specific binding sites of enzymes and inhibiting their activities (Bücker-Neto et al., 2017; Jalmi et al., 2018). The resulting Ni stress–induced oxidative stress damages plant biomolecules and impairs their antioxidant defense systems (Gajewska and Skłodowska, 2005; Ghasemi et al., 2009; Huang et al., 2019).

Phytohormones play pivotal roles in regulating plant growth and development, as well as stress tolerance (Zhao et al., 2021). Because the toxic levels of heavy metals induce adverse effects to plant growth and physiology including low biomass, chlorosis, photosynthesis inhibition, altered water and nutrient balance, and senescence, phytohormones have the potential to alleviate metal toxicity in plants and rescue metal toxicity–induced growth retardation (Singh and Prasad, 2016; Bücker-Neto et al., 2017; Sytar et al., 2019). Heavy metal stress extensively alters the expression of genes involved in the biosynthesis, transport, conjugation, and response of phytohormones including auxin (indole-3-acetic acid, IAA), cytokinin (CK), gibberellin (GA), ethylene (ETH), abscisic acid (ABA), brassinosteroid (BR), salicylinc acid (SA), and jasmonic acid (JA) (Saini et al., 2021). Different classes of phytohormones mitigate heavy metal stress and toxicity in different ways (Bücker-Neto et al., 2017; Jalmi et al., 2018; Sytar et al., 2019). Heavy metal stress such as Cd and As negatively regulates transcription of auxin-related genes involved in biosynthesis and transport and perturbs the auxin homeostasis resulting in growth defects (Fattorini et al., 2017; Luo et al., 2019). Auxin is one of the most multi-functional phytohormones regulating plant development and stress responses (Teale et al., 2006; Vanneste and Friml, 2009; Bielach et al., 2017); However, various heavy metals reduce auxin accumulation and distribution, suggesting that auxin homeostasis is essential for heavy metal stress tolerance (Wang et al., 2015; Bücker-Neto et al., 2017). In addition, transcripts of auxin homeostasis–related genes, including those involved in the biosynthesis, transport, degradation, and signaling of this phytohormone, displayed different responses to heavy metals in the model plant *Arabidopsis thaliana*, for example, they were up-regulated in the shoots of Arabidopsis seedlings under Ni stress, but not in response to other heavy metal stresses (Wang et al., 2015). In addition, exogenous auxin applications alleviate the toxicity of heavy metal stresses caused by Pb, As, and Cd (Bücker-Neto et al., 2017), but this response has not yet been explored for Ni stress.

The natural auxin IAA is generated from IAA precursors, including indole-3-acetonitrile (IAN), indole-3-acetaldehyde (IAAld), indole-3-acetamide (IAM), indole-3-acetaldoxime (IAOx), and indole-3-pyruvic acid (IPA) (Enders and Strader, 2015). The IPA pathway is considered to be the major auxin biosynthesis route, involving a two-step conversion of tryptophan (Trp) to IAA driven by the enzymatic reaction of the TAA family of aminotransferases (TAA) and subsequent YUCCA (YUC) activity (Zhao, 2012). The YUC subfamily belongs to the flavin monooxygenase (FMO) family, with YUC1 first being identified as a key auxin biosynthesis enzyme in a rate-limiting step (Zhao et al., 2001). We previously reported that Arabidopsis YUC6 also functions as a thiol-reductase (TR), reducing toxic ROS levels, promoting oxidative and drought stress tolerance, and delaying leaf senescence (Cha et al., 2015; Cha et al., 2016). In addition, we found that the Cys85 residue on YUC6 is essential for its TR activity but not its FMO activity, which regulates its antioxidant enzyme activity and the transcription of the auxin efflux carrier *PINFORMED* (*PIN*) genes (Cha et al., 2015; Cha et al., 2016).

Here, we examined whether an exogenous auxin application reduces Ni heavy metal stress toxicity, and whether the TR activity of YUC6 confers Ni stress tolerance by inhibiting the accumulation of toxic ROS in Arabidopsis.

## Materials and methods

### Plant material and growth conditions

Arabidopsis (*Arabidopsis thaliana*) plant materials including wild-type (Col-0 and Col-gl), *yuc6-1D* (Col-gl ecotype background), YUC6-, YUC6^C85S^- and CYP79B2-overexpressing transgenic plants (Col-0 ecotype background) were used in previous publications (Kim et al., 2007; Cha et al., 2015). Seeds were surface sterilized with 30% bleach for 5 min, cold-treated for 2 days at 4°C, and sown on a half-strength Murashige and Skoog (MS) agar medium. Plants were grown at 23°C under a 16 h/8 h light-dark cycle in a growth chamber.

### Ni stress treatments

Seeds were sown on a half-strength MS agar medium supplemented with different concentrations of Ni (as NiCl_2_). To examine the exogenous application of IAA on Ni stress response, different concentrations of IAA were additionally supplemented with Ni-containing media. To examine the effects of YUC-induced auxin biosynthesis on Ni stress response, a potent YUC inhibitor YUCASIN [5–(4–chlorophenyl)-4H-1,2,4–triazole-3–thiol, Wako (Nishimura et al., 2014)] was dissolved in dimethyl sulfoxide as a stock solution (50 mM, stored at -20°C until use) and further diluted to be 125 µM YUCASIN in Ni-containing media. Plants were grown on the media mentioned above for 2.5 weeks and used for measuring physiological parameters including fresh weight and chlorophyll contents. Plants were treated with 100 µM Ni [a concentration inducing transcriptional, physiological, and morphological changes (Le¡ková et al., 2017; Lešková et al., 2020)] for Inductively Coupled Plasma-Mass Spectrometer (ICP-MS), malondialdehyde (MDA) assay, histochemical staining, glutathione assay, antioxidant enzyme assay, and real-time quantitative PCR (qPCR) analysis.

### Chlorophyll contents

Chlorophyll concentrations were determined by extracting shoot samples with 80% (v/v) acetone at 23°C for 48 h in the dark. Chlorophyll a and b were measured at 663 and 645 nm, respectively, and total chlorophyll contents were shown as milligrams of chlorophyll per gram of fresh leaves (Ni et al., 2009).

### Ion concentrations by ICP-MS

Three-week-old plants grown on MS medium were exposed to 0 (as deionized H_2_O) or 100 µM Ni for 1 day under 16 h light/8 h dark cycle at 23°C. Plants were rinsed with deionized water and dried at 65°C for 2 days. The dried samples were ground using a mortar and pestle, and 100 mg of samples were extracted in 10 ml of HCIO_4_:H_2_O: H_2_SO_4_ (9:5:1, v/v/v) on a heating block with a gradual increase in temperature from 100°C to 320°C until the digestion was completed. The diluted supernatant (1:10 ratio into the deionized water) was filtered and submitted to an ICP-MS (Perkin Elmer Optima 2200 DV) to quantify the concentrations of mineral elements as previously reported (Jeon et al., 2019).

### MDA assay and H_2_O_2_ quantification

Two-week-old plants grown on MS medium were treated with or without 100 µM Ni for 12 hrs and rinsed with deionized water. The leaf tissue was frozen immediately and ground using liquid nitrogen. Samples (0.1 g) were homogenized in 1.5 ml of reaction buffer containing 20% (w/v) trichloric acid (TCA) and 0.5% (w/v) thiobarbituric acid (TBA) and vortex for 1 min. The homogenates were centrifuged at 13,000 rpm for 15 min at 4°C, and supernatants were heated at 85°C for 20 min. After incubation on ice for 5 min, absorbance in supernatants was measured at 532 and 600 nm. MDA contents were calculated using an extinction coefficient of 155 mM^-1^ cm^-1^ as described previously (Wang et al., 2019). For measuring H_2_O_2_ contents, plant tissues were extracted in 20 mM potassium phosphate buffer (pH 6.5) and rescued the supernatants after centrifugation at 13,000 rpm for 15 min at 4°C. The H_2_O_2_ contents were determined using the Amplex Red Hydrogen Peroxide/Peroxidase Assay Kit (Invitrogen) according to the manufacturer’s instructions (Cha et al., 2015).

### Histochemical staining for superoxide anion and hydrogen peroxide

Two-week-old plants were treated with or without 100 µM Ni for 8 hrs and immediately stained with 0.1% (w/v, pH 7.8) nitro blue tetrazolium (NBT, Sigma Aldrich) and 1 mg ml^-1^ (pH 3.8) 3,3’-diaminobenzidine (DAB, Sigma Aldrich). Chlorophylls in the NBT and DAB-stained leaves were removed by subsequent incubation in 80% (v/v) ethanol. Six-day-old seedlings were treated with or without 100 µM Ni for 8 hrs and immediately submerged in 10 µM 2’,7’-dichlorodihydrofluorescein diacetate (H_2_DCF-DA, Thermo Fisher Scientific). Seedlings were then washed with 10 mM MES, 0.1 mM KCl, and CaCl_2_ (pH 6.0) and were incubated for 1 h at 22°C. Fluorescence signals (excitation 488 nm and emission 522 nm) were monitored using confocal microscopy (Olympus).

### Measurements of reduced (GSH) and oxidized (GSSG) glutathione contents

Two-week-old plants were treated with or without 100 µM Ni for 12 hrs and frozen immediately and ground using liquid nitrogen. GSH and GSSG contents in samples were analyzed using Glutathione (GSH/GSSG/Total) Assay Kit (OxiTec™) following the manufacturer’s protocol at 412 nm.

### Antioxidant enzyme assay

The supernatants extracted for H_2_O_2_ measurements were analyzed for peroxidase (PRX) and catalase (CAT) activity using an Amplex Red Hydrogen Peroxide/Peroxidase Assay Kit and Amplex Red Catalase Assay Kit (Invitrogen), respectively, according to the manufacturer’s instructions. Fluorescence (excitation 530 nm and emission 590 nm) was detected by a spectrofluorometer (Molecular Device). Superoxide dismutase (SOD) activity was measured using an SOD Determination Kit (Sigma-Aldrich) according to the manufacturer’s instructions. Thiol peroxidase (thiol PRX) activity was spectrophotometrically monitored by NADPH consumption at 340 nm in the presence of 100 mM Tris-Cl (pH 8), 0.3 mM NADPH, 0.8 µM recombinant Arabidopsis Thioredoxin (Trx)-h2, 0.18 µM recombinant Arabidopsis NADPH-dependent Trx reductase A (NTRA), and 100 µM H_2_O_2_ as described previously with slight modification (Delaunay et al., 2002).

### Transcriptional analysis

Two-week-old plants were treated with or without 100 µM Ni for 6 hrs. The total RNA of plants was extracted with Total RNA Prep Kit (BioFACT) according to the manufacturer’s instructions. The cDNA templates were synthesized using RevertAid First Strand cDNA Synthesis Kit (Thermo Fisher Scientific). Synthesized cDNA was amplified by qPCR with CFX96 Touch Real-Time PCR Detection System (Biorad) using TOPreal™ qPCR 2X PreMIX (SYBR Green with low ROX/Enzynomics). The expression level of target genes was normalized to that of the housekeeping gene, *AT5G12240 or Tubulin 2* (*TUB2*)*, via* the delta-delta Ct (ΔΔCt) method. All primers used for qPCR are listed in **Supplemental Table 1**.

## Results

### Exogenous auxin applications confer Ni stress tolerance in Arabidopsis

**Figure 1:**
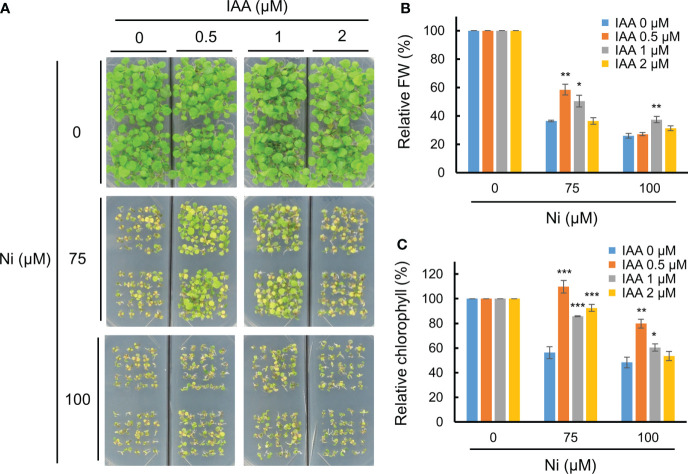
Exogenous auxin partially enhanced Ni stress tolerance. **(A)** Phenotypes of wild-type (WT, Col-0) plants subjected to various Ni concentrations combined with different IAA concentrations. WT plants were grown in a half-strength MS medium containing different concentrations of Ni and IAA and photographed at 2.5 weeks after germination. Relative fresh weight (FW, **B**) and relative chlorophyll contents **(C)** in leaves of plants exposed to 0, 75, or 100 μM Ni with or without 0, 0.5, 1, and 2 μM IAA for 2.5 weeks. Values are means ± SE of three independent biological replicates. Asterisks indicate significant differences (*p < 0.05, **p < 0.01, and ***p < 0.001) from the 0 µM IAA treatments at each Ni concentration determined using two-tailed Student’s *t*-test.

A previous report using a GUS reporter line driven by the *DR5* promoter containing an auxin-responsive TGTCTC element showed that various heavy metal stresses differently alter auxin accumulation and distribution in shoots and roots of Arabidopsis, and Ni stress reduces auxin levels in both parts (Wang et al., 2015). This suggests that heavy metal stress disrupts auxin homeostasis in plants, and that an exogenous auxin application could rescue heavy metal toxicity. We therefore investigated the growth retardation of Arabidopsis WT (Col-0) plants under various Ni concentrations combined with different IAA applications. In the absence of IAA (0 µM), Ni stress dramatically inhibited the growth of WT plants both under 75 and 100 µM Ni stress conditions (**Figure 1A**). The growth retardation under Ni stress was rescued by adding an exogenous application of 0.5 or 1 µM IAA, although 2 µM IAA treatments did not restore the phenotype (**Figure 1A**). The lower relative fresh weights and chlorophyll contents under Ni stress conditions were also significantly increased by the addition of 0.5 or 1 µM IAA (**Figures 1B, C**). Auxin is known to be a versatile regulator that differentially influences plant growth and development depending on its concentration; low auxin concentrations promote root and shoot growth by inducing cell enlargement, whereas high concentrations repress growth (Sauer et al., 2013; Dünser and Kleine-Vehn, 2015). In addition, we confirmed whether exogenous IAA treatments change the other stress-related phytohormones such as ABA and ETH through the transcriptional analysis of their responsive genes, such as *ABA INSENSITIVE 4* (*ABI4*) and *ETHYLENE INSENSITIVE 3* (*EIN3*), respectively (Potuschak et al., 2003; Shkolnik-Inbar and Bar-Zvi, 2010). However, the expressions of both genes (*ABI4* and *EIN3*) did not significantly change by exogenous treatment of 1 µM IAA (**Supplementary Figure 1**). These findings demonstrate that exogenous auxin applications below a concentration of 1 µM reduce Ni heavy metal toxicity in Arabidopsis.

### Dominant mutant *yuc6-1D* shows Ni stress tolerance

**Figure 2:**
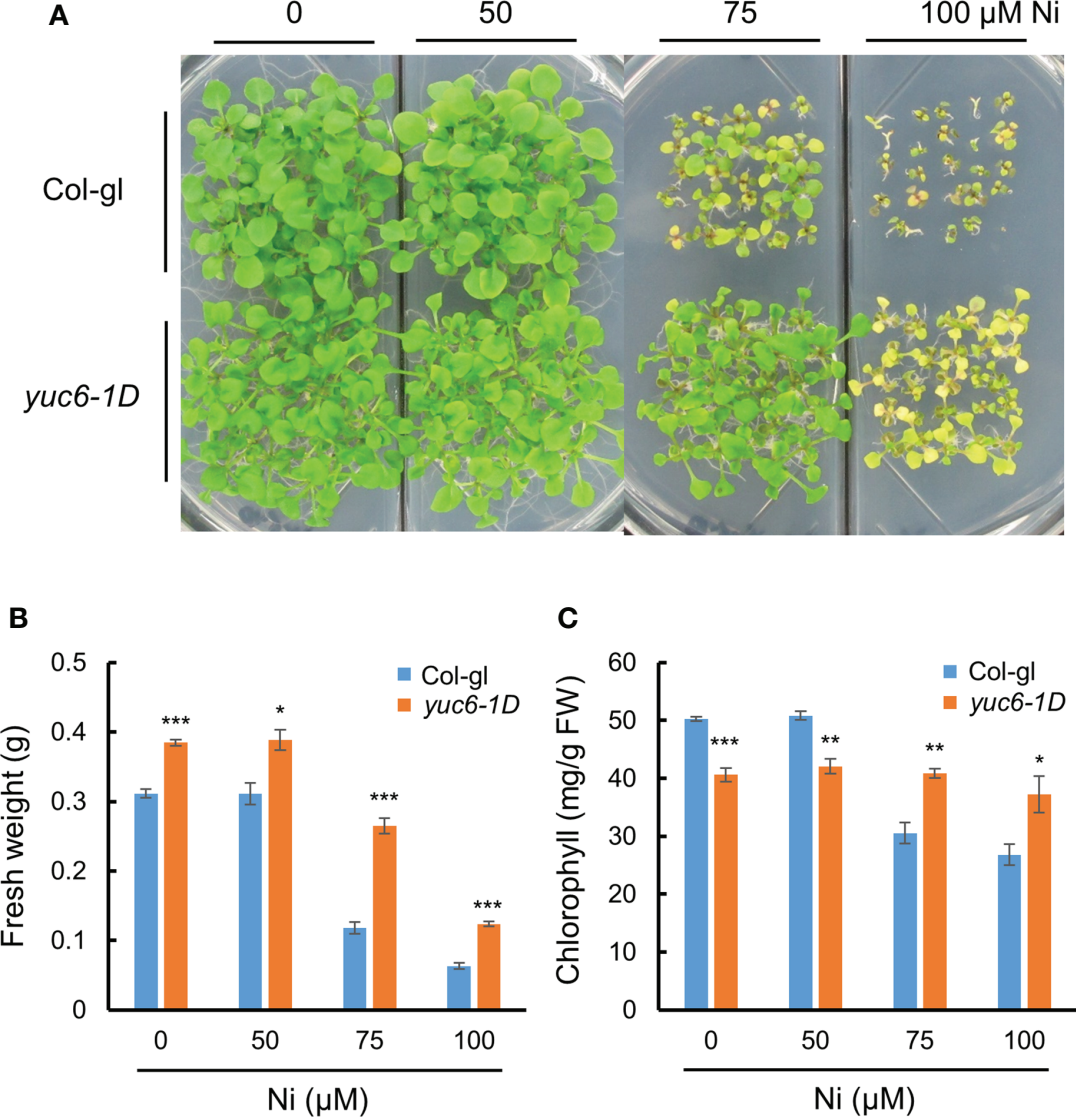
Dominant mutant *yuc6-1D* shows Ni stress tolerance. Phenotypes **(A)**, fresh weight **(B)**, and chlorophyll contents **(C)** of wild-type (WT, Col-gl) and *yuc6-1D* plants grown on 0, 50, 75, or 100 μM Ni for 2.5 weeks after germination. Values are means ± SE of three independent biological replicates. Asterisks indicate significant differences (*p < 0.05, **p < 0.01, and ***p < 0.001) from the WT determined using two-tailed Student’s *t*-test.

Next, we considered whether elevated endogenous auxin levels could also mediate Ni stress tolerance in Arabidopsis. The 11 YUC members in Arabidopsis are auxin biosynthesis enzymes that directly convert IPA to IAA (Schlaich, 2007; Zhao, 2012). We previously showed that the dominant Arabidopsis mutant of *YUC6*, *yuc6-1D*, displayed the unique phenotypes of auxin overproduction including epinastic cotyledons, increased apical dominance, and curled leaves, which phenocopied the activation tagging mutant *yuc1* (Zhao et al., 2001; Kim et al., 2007). Here, we compared the Ni stress response of *yuc6-1D* plants against its WT background, Col-gl, revealing that *yuc6-1D* was much more tolerant of all examined Ni concentrations than the WT (**Figure 2A**). In addition, the fresh weights and chlorophyll contents of the *yuc6-1D* plants were significantly higher than Col-gl under all Ni stress conditions tested (**Figures 2B, C**). Previous work has shown that the free auxin levels of *yuc6-1D* were 25% higher than those of Col-gl (Kim et al., 2007), which, together with our results, suggests that the elevated endogenous auxin levels in *yuc6-1D* could reduce Ni stress toxicity.

### *yuc6-1D* absorbs more Ni than WT Arabidopsis

**Figure 3:**
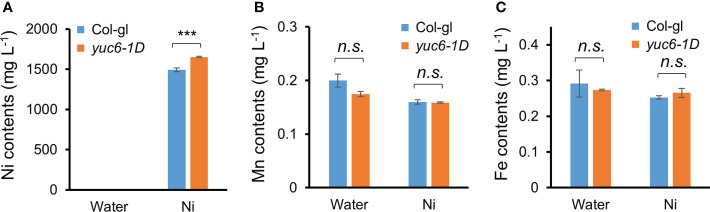
Dominant mutant *yuc6-1D* accumulates higher Ni ions compared to WT. Three-week-old Col-gl WT and *yuc6-1D* plants were treated with 100 µM Ni for 1 day. Nickel **(A)**, Manganese **(B)**, and Iron **(C)** contents were measured by an ICP-MS. Values are means ± SE of three independent biological replicates. Asterisks indicate significant differences (***p < 0.001; *n.s.*, no significant) from the WT determined using the two-tailed Student’s *t*-test.

To elucidate the reason behind the enhanced Ni stress tolerance of *yuc6-1D* plants, we measured their Ni and other ion contents using an ICP-MS analysis. Three-week-old Col-gl and *yuc6-1D* plants were exposed to 0 or 100 µM Ni for one day, after which the concentration of 23 cations were measured using ICP-MS. Although the Ni contents were dramatically increased in the Ni treated plants of both genotypes, *yuc6-1D* contained significantly more (10.5% more) Ni than Col-gl (**Figure 3A**). Ni treatments reduce manganese (Mn) and iron (Fe) availability for plants, both of which are necessary for chlorophyll biosynthesis and photosynthesis (Rahman et al., 2005; Fasani et al., 2021; Rai et al., 2021). Our data showed that Ni stress decreased the Mn and Fe contents to a similar extent in Col-gl and *yuc6-1D* (**Figures 3B, C**). No significant differences were observed in the contents of the other cations between the treatments and/or genotypes (**Supplementary Table 2**). These results indicate that Ni stress tolerance in *yuc6-1D* plants is not caused by the inhibition of Ni absorption or competing cations.

### The TR activity of YUC6 is essential for Ni stress tolerance

**Figure 4:**
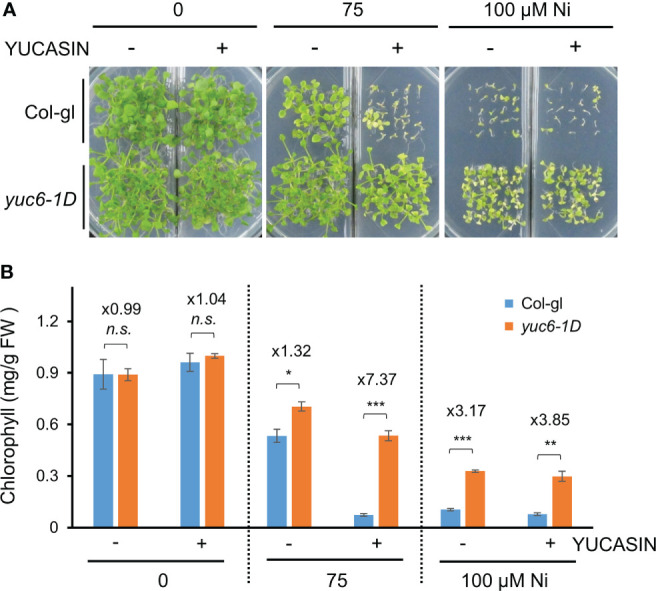
The *yuc6-1D* mutant displays Ni stress tolerance independent of IAA biosynthesis. Phenotypes **(A)** and chlorophyll contents **(B)** of Col-gl WT and *yuc6-1D* plants grown in 0, 75, or 100 μM Ni-containing media in the absence or presence of 125 µM YUCASIN. Values are means ± SE of three independent biological replicates. Asterisks indicate significant differences (*p < 0.05, **p < 0.01, ***p < 0.001, and *n.s.*, no significant) from the WT determined using two-tailed Student’s *t*-test. The numbers above the bars indicate the ratio of chlorophyll contents of WT and *yuc6-1D* (Col-gl and *yuc6-1D*).

YUCASIN was previously shown to specifically inhibit the ability of YUC to produce IAA *in vitro* and *planta*, suggesting that YUCASIN is a potent inhibitor of IAA biosynthesis (Nishimura et al., 2014). In addition, YUCASIN suppressed IAA overproduction in *YUC1*-OX plants and reduced IAA levels as WT (Nishimura et al., 2014). We therefore investigated whether the reduction of IAA levels by YUCASIN affected the Ni stress toxicity in Arabidopsis. The Ni stress–induced growth retardation in the WT (Col-gl) plants was dramatically worsened by YUCASIN, suggesting that the maintenance of endogenous auxin levels is necessary for Ni stress tolerance (**Figure 4A**). In addition, *yuc6-1D* plants under Ni stress were less sensitive to YUCASIN (**Figure 4A**). The Ni stress toxicity (75 µM Ni) of Col-gl was strongly affected by YUCASIN, as shown by the 86.5% reduction in chlorophyll in the Ni-stressed plants when treated with YUCASIN. By contrast, the chlorophyll content of Ni-stressed *yuc6-1D* plants was only reduced by 24.1% when subjected to YUCASIN (**Figure 4B**). Ni stress altered transcription of various auxin biosynthetic genes including *TAA1* (IPA pathway), *cytochrome P450* gene *CYP79B2* (IAOx pathway), *AMIDASE 1* (*AMI1*, IAM pathway), and *NITRILASE 1* (*NIT1*, IAN pathway), but they did not correlated with Ni-stress tolerance in *yuc6-1D* (**Supplementary Figure 2**). These data suggest that YUC6 possesses another biochemical activity that enables Arabidopsis to endure Ni heavy metal stress rather than IAA biosynthetic activity.

**Figure 5:**
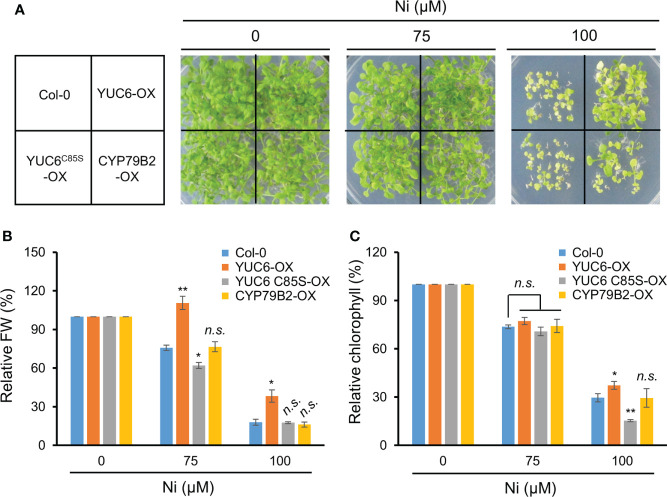
TR activity of YUC6 is necessary for YUC6-acquired Ni stress tolerance. Phenotypes **(A)**, relative fresh weight **(B)**, and relative chlorophyll contents **(C)**. Wild-type (Col-0), YUC6-OX, YUC6^C85S^-OX, and CYP79B2-OX plants were grown in a half-strength MS medium containing 0, 75, or 100 μM Ni for 2.5 weeks. Values are means ± SE of three independent biological replicates. Asterisks indicate significant differences (*p < 0.05, **p < 0.01, and *n.s.*, no significant) from the WT at each Ni concentration determined by two-tailed Student’s *t*-test.

Our previous studies demonstrated that Arabidopsis YUC6 has a dual function as an FMO-like and TR enzyme for IAA biosynthesis and ROS reduction, respectively (Cha et al., 2015). The TR activity of YUC6 requires an essential cysteine at residue 85 (Cys85), which mediates drought tolerance and delayed leaf senescence independently of auxin biosynthesis (Cha et al., 2015; Cha et al., 2016). We previously generated 35S promoter–driven OX lines of YUC6, YUC6^C85S^ (containing a point mutation of Cys85 to Ser), and CYP79B2, all of which displayed high IAA levels compared with the WT Col-0 but no significant difference between the YUC6-OX, YUC6^C85S^-OX, and CYP79B2-OX plants (Cha et al., 2015). Based on these findings, we examined whether the TR activity of YUC6 is necessary for Ni stress tolerance. The YUC6-OX plants were more tolerant of 100 µM Ni stress conditions than the WT, whereas the YUC6^C85S^-OX plants were affected to a similar level as Col-0 (**Figure 5A**). CYP79B2-OX plants showed slightly better growth than Col-0 and YUC6^C85S^-OX plants under the stressed conditions (**Figure 5A**). The relative fresh weight and chlorophyll contents of YUC6-OX were significantly higher than Col-0 under the 75 and 100 µM Ni stress conditions, while they were significantly lower for YUC6^C85S^-OX under 75 and 100 µM Ni, respectively (**Figures 5B, C**); neither physiological parameter was significantly different in the CYP79B2-OX plants. The TR activity of YUC6 *via* Cys85 is therefore essential for YUC6-mediated Ni stress tolerance in Arabidopsis.

### YUC6 inhibits Ni stress–induced oxidative damage

**Figure 6:**
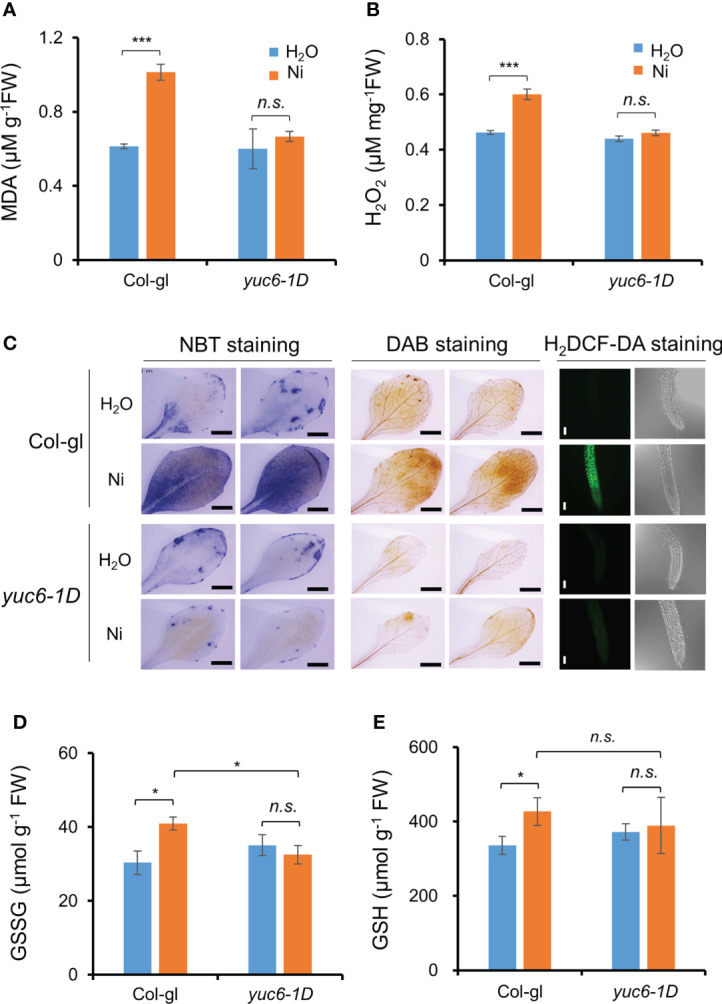
YUC6 inhibits Ni stress-induced oxidative damage *via* inhibition of ROS accumulation. Two-week-old Col-gl WT and *yuc6-1D* plants were treated with or without 100 μM Ni for 12 hrs and measured MDA **(A)**, H_2_O_2_ **(B)**, GSH **(D)**, and GSSG contents **(E)**. Values are means ± SE of three **(A, B)** or four **(D, E)** independent biological replicates. Asterisks indicate significant differences (*p < 0.05, ***p < 0.001, and *n.s.*, no significant) from H_2_O treatments in each genotype or between Ni-treated Col-gl and *yuc6-1D* determined by a two-tailed Student’s *t*-test. **(C)** Histochemical staining for detecting Ni stress-induced ROS accumulation in plant cells. Six-day-old seedlings stained by NBT, DAB, and H_2_DCF-DA. Representative images are shown from 3 independent experiments. Scale bar = 1 mm (for NBT and DAB) or 100 µm (H_2_DCF-DA).

Our previous report showed that *yuc6-1D* accumulated less ROS under drought stress and an oxidative stress agent, methyl viologen (Cha et al., 2015). Various heavy metal stresses lead to ROS accumulation, causing cellular oxidative stress (Schützendübel and Polle, 2002; Sytar et al., 2013; Sytar et al., 2019). We therefore measured lipid peroxidation to determine whether Ni stress–induced oxidative damage is reduced in *yuc6-1D*. The MDA content of *yuc6-1D* did not differ in plants exposed to the H_2_O and Ni stress treatments, whereas the MDA content of Col-gl was significantly increased by Ni stress (**Figure 6A**). This suggests that YUC6 inhibits the oxidative damage caused by Ni stress.

We further explored whether the lower MDA content of *yuc6-1D* is the result of lower ROS levels. First, we measured the endogenous levels of a predominant toxic radical, H_2_O_2_, in plants. The H_2_O_2_ content of Col-gl was significantly increased by Ni stress, while this was not the case in *yuc6-1D* is not, consistent with the MDA contents (**Figure 6B**). In addition, the H_2_O_2_ content of YUC6-OX plants was not significantly affected by Ni stress treatment, while those of the Col-0 WT and the YUC6^C85S^-OX plants were significantly elevated (**Supplementary Figure 3**). This suggests that the TR activity of YUC6 is necessary for alleviating Ni stress–induced ROS accumulation. Next, we visualized the endogenous ROS accumulation in plant tissues using histochemical staining. NBT and DAB stains are widely used to detect endogenous superoxide anion (O_2_˙¯) and H_2_O_2_, respectively (Chae et al., 2021). Both radicals accumulated to high levels in Col-gl under Ni stress; however, in *yuc6-1D* plants their levels were similar in both the control and Ni stress conditions (**Figure 6C**). In addition, the fluorescence signals of H_2_DCF-DA, a ROS-sensing fluorescent dye, were enhanced by Ni stress in Col-gl; however, this signal was not detected in *yuc6-1D* under either the control or Ni–stressed conditions (**Figure 6C**). YUC6 therefore negatively regulates Ni stress–induced oxidative damage through the alleviation of toxic ROS.

Glutathione is an essential cellular component for the defense system in plants exposed to stress conditions and plays as a metal chelator and antioxidant to scavenge the toxic ROS (Jozefczak et al., 2012; Hameed et al., 2014). The glutathione exists as oxidized (GSSG) and reduced (GSH) forms cycling *via* thiol–disulfide interchange reactions, and stress–induced GSSG accumulation is biochemical marker for oxidative stress (Rahantaniaina et al., 2013). Thus, we measured GSSG and GSH contents in Ni–exposed plants. GSSG contents in Col-gl WT plants were significantly increased by Ni stress, whereas those in *yuc6-1D* plants did not change (**Figure 6D**). Elevated GSSG contents in WT by Ni stress were also significantly higher than *yuc6-1D*, which is consistent with less oxidative damage in *yuc6-1D* (**Figures 6A–C**), but GSH contents under Ni stress conditions were not different between WT and *yuc6-1D* plants (**Figures 6D, E**). It suggests that Ni stress tolerance of *yuc6-1D* is independent of GSH levels.

### YUC6 enhances the PRX activity and transcription of *PrxQ*

**Figure 7:**
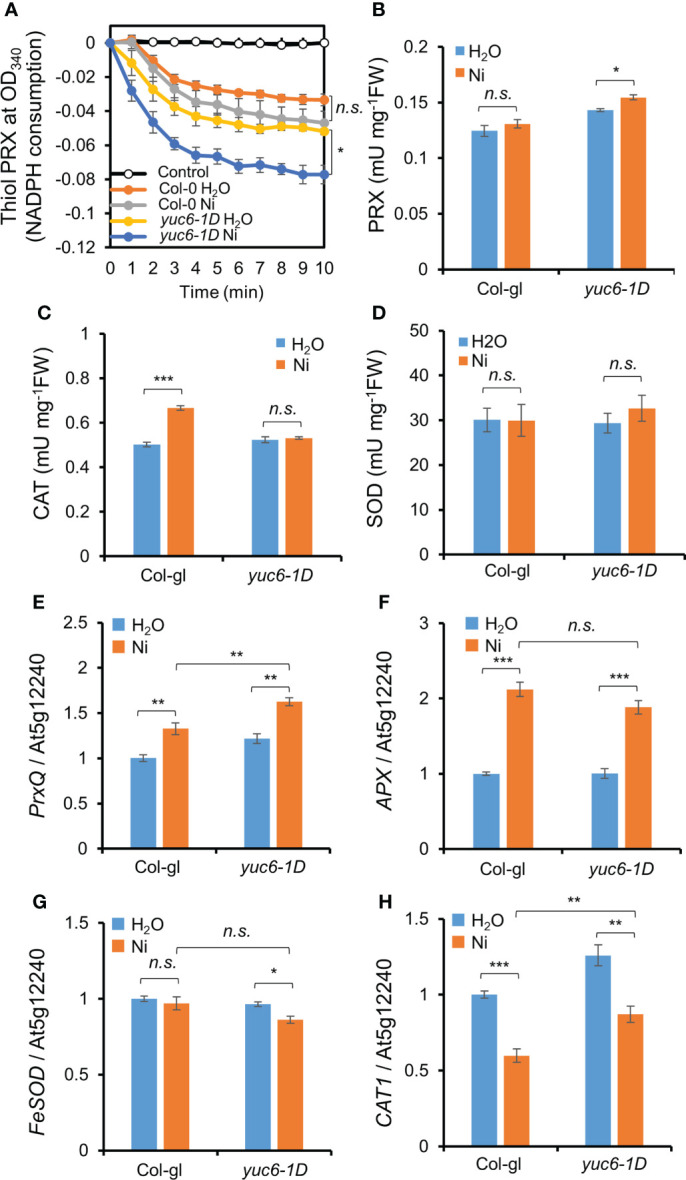
YUC6 positively regulates thiol PRX/PRX enzyme activities and *PrxQ* transcription. Two-week-old Col-gl WT and *yuc6-1D* plants were treated with or without 100 μM Ni for 12 hrs (for antioxidant enzyme activities, **A–D**) or 6 hrs (for qPCR, **E–H**). Thiol PRX **(A)**, PRX **(B)**, CAT **(C)**, and SOD **(D)** activities. Thiol PRX **(A)** was measured by the NADPH consumption at OD_340_ in the presence of AtTRX-h2, AtNTRA, H_2_O_2_, and NADPH. Total proteins extracted from Col-gl and *yuc6-1D* with or without Ni stress were applied to the thiol PRX assay. Control is monitored without plant extracts. PRX **(B)**, CAT **(C)**, and SOD **(D)** activities were monitored using the plant extracts mentioned above. Transcription of *PrxQ* **(E)**, *APX* **(F)**, *FeSOD* **(G)**, and *CAT1* **(H)**. The expression levels of antioxidant genes **(E–H)** were normalized to *AT5G12240*. Values are means ± SE of three **(A–C, E–H)** or four **(D)** independent biological replicates. Asterisks indicate significant differences (*p < 0.05, **p < 0.01, ***p < 0.001, and *n.s.*, no significant) from the H_2_O treatments in each genotype or between Ni-treated Col-gl and *yuc6-1D* determined by two-tailed Student’s *t*-test.

Antioxidant enzymes efficiently scavenge toxic ROS molecules (Miller et al., 2010). We previously reported that the PRX activity in *yuc6-1D* and YUC6-OX plants was enhanced in the absence of stress and that this activity is regulated by the TR activity of YUC6 (Cha et al., 2015). We therefore examined the antioxidant enzyme activities in the absence or presence of Ni stress. The already enhanced thiol PRX and total PRX activities in *yuc6-1D* in the absence of Ni stress were significantly elevated under Ni stress conditions (**Figures 7A, B**). By contrast, the CAT activity in *yuc6-1D* did not significantly differ between the control and Ni stress conditions, while the CAT activity in Col-gl plants was significantly enhanced by Ni stress (**Figure 7C**). SOD activity also did not significantly differ between genotypes and treatments (**Figure 7D**). Enhanced PRX antioxidant enzyme activity under control conditions was consistent with our previous results (Cha et al., 2015). These findings suggest that YUC6 positively regulates PRX activity including thiol PRX, enhancing the antioxidant capacity and conferring tolerance to Ni heavy metal stress.

Next, we examined the transcriptional levels of various antioxidant genes. Ni stress activated the transcription of *peroxiredoxin Q* (*PrxQ*) and *ascorbate peroxidase* (*APX*) in both Col-gl and *yuc6-1D* (**Figures 7E, F**). The *PrxQ* expression was significantly higher in *yuc6-1D* than in Col-gl in the absence of Ni stress, and its expression was more strongly enhanced in *yuc6-1D* by Ni stress (**Figure 7E**). The expression levels of *APX*, *iron superoxide dismutase* (*FeSOD*), and *catalase 1* (*CAT1*) in *yuc6-1D* were not significantly enhanced by Ni stress (**Figures 7F–H**). PRXs are ubiquitous, and the highly conserved cysteine-dependent thiol PRX family are known to be responsible for the removal of more than 90% of cellular peroxide (Perkins et al., 2015). In addition, it has been revealed that PrxQ catalytically removes peroxides by receiving electrons from thioredoxin (TRX)/TRX reductase systems (Su et al., 2018), and is correlated with enhanced thiol PRX activity *in yuc6-1D* (**Figure 7A**). Thus, the TR activity of YUC6 may activate the transcription of *PrxQ* and/or donate electrons to PrxQ, which scavenges toxic ROS molecules under Ni stress conditions.

## Discussion

As a consequence of anthropogenic activities, such as urbanization and industrialization, climates and soil environments are seriously deteriorating, both of which are harmful to crop production (Okereafor et al., 2020). Heavy metals are non-biodegradable major soil contaminants limiting plant growth and crop productivity (Tiwari and Lata, 2018; Ghori et al., 2019). Heavy metal stress activates various signaling networks in plants, such as the calcium, MAPK, and hormone signaling pathways (Jalmi et al., 2018). Endogenous molecules called phytohormones play pivotal roles in most aspects of plant growth and development, as well as stress responses (Zhao et al., 2021). Due to these biological effects, exogenous applications of phytohormones are extensively applied in agriculture to maintain and increase crop productivity despite unfavorable environmental changes (Egamberdieva et al., 2017). Recently, phytohormones have been adapted in a promising technique by which plants can restore heavy metal contaminated soils (Sytar et al., 2019). Heavy metal stresses inhibit seed germination and root and shoot growth, all of which are mainly controlled by phytohormones (Sytar et al., 2019). Various heavy metals, such as Cd, Hg, Cu, increase the endogenous abscisic acid (ABA) levels due to the transcriptional activation of ABA biosynthesis genes (Bücker-Neto et al., 2017; Sytar et al., 2019). Cytokinins (CKs) positively regulate the phytoextraction of Pb and Zn by stimulating cell division and shoot initiation in sunflower (*Helianthus annuus*) (Tassi et al., 2008), while gibberellin (GA) treatments alleviate Cd toxicity by reducing the accumulation of nitric oxide (NO) in Arabidopsis (Zhu et al., 2012). Exogenous IAA applications help to maintain plant growth and biomass under Pb, Zn, and Cd in various plants, such as sunflower, maize (*Zea mays*), switchgrass (*Panicum virgatum*), and wheat (*Triticum aestivum*) (Sytar et al., 2019). Endogenous IAA levels were found to be affected by various heavy metal stresses, restricting the homeostasis and distribution of this phytohormone in Arabidopsis (Wang et al., 2015). Interestingly, the transcription levels of the IAA homeostatic genes, including those involved in biosynthesis, conjugation, and degradation were highly up-regulated in the shoots under Ni stress conditions but down–regulated in the roots, whereas other heavy metals differently regulated their expressions (Wang et al., 2015). Thus, Ni stress may be a major heavy metal stress limiting proper plant growth *via* IAA homeostasis. The results of our present study showed that exogenous applications of IAA reduced Ni stress–induced growth retardation in Arabidopsis, although IAA treatments above 2 µM did not have this beneficial effect (**Figure 1**). Plant growth, including both the shoots and roots, is known to be differently regulated depending on IAA concentrations; low concentrations promote growth, while high concentrations repress growth (Dünser and Kleine-Vehn, 2015). In addition, a treatment of YUCASIN, a potent inhibitor of YUC–induced auxin biosynthesis, enhanced Ni stress sensitivity (**Figure 4**), providing further evidence that the maintenance of endogeneous auxin levels is necessary for plants to endure Ni stress toxicity.

Like many other environmental stresses, heavy metals cause oxidative stress *via* toxic ROS accumulation and the subsequent impairing of the antioxidant system in plant cells (Miller et al., 2010; Ghori et al., 2019). At optimum levels, ROS function as essential signaling molecules for plant development, but can be toxic when present in excess, inducing oxidative damage to biomolecules such as DNA, proteins, and lipids (Miller et al., 2010; Mittler, 2017). Various heavy metal stresses modulate all aspects of IAA metabolism and dynamic polar transport (Wang et al., 2015). It has been reported that treatments of a glutathione biosynthesis inhibitor (buthionine sulfoximine, BSO) decrease the expression of genes encoding the auxin efflux transporters, known as PINs, in Arabidopsis (Bashandy et al., 2010; Koprivova et al., 2010). We also have identified that treatments of BSO or the ROS generator methyl viologen reduced the expression of the *PIN*s (Cha et al., 2016), suggesting that a ROS imbalance in plant cells impairs the auxin availability by decreasing the abundance of auxin efflux transporters. Stress-induced ROS is scavenged by various antioxidant enzymes, such as the SODs, CATs, PRXs, TRXs, and glutaredoxins (GRXs) (Dumanović et al., 2021). Interestingly, the dysfunction of NADPH-dependent TRX reductase (NTR) and GRXs systems in Arabidopsis induced defects in various auxin signaling–mediated developmental processes, such as a loss of apical dominance, vasculature defects, and reduced secondary root production, suggesting that interplay between the NTR and GRX systems plays a major role in auxin homeostasis (Bashandy et al., 2010; Koprivova et al., 2010). We previously reported that the auxin biosynthesis enzyme YUC6 possesses TR activity *in vitro*, and plants overexpressing *YUC6* or those containing the dominant mutation *yuc6-1D* accumulate less ROS both in the absence and presence of oxidative stress (Kim et al., 2013; Cha et al., 2015). In addition, *YUC6*-OX Arabidopsis and potato (*Solanum tuberosum*) plants, as well as *yuc6-1D* plants, showed greater expression levels in various antioxidant genes, including those encoding CuZnSOD, APX, CAT2, PRX, and various oxidoreductases (Kim et al., 2013; Cha et al., 2015). Thus, this biochemical and molecular evidence indicated that YUC6 could reduce heavy metal stress–induced oxidative stress. Our data showed that *yuc6-1D* and YUC6-OX plants were tolerant of Ni stress, displaying less oxidative damage due to their restriction of ROS accumulation under Ni stress conditions (**Figures 2**, **5**, **6**). NTRs in land plants have two conserved neighboring catalytic cysteines (CXXC) in a redox-active site (Jacquot et al., 2009). We previously identified that a Cys85 residue in YUC6 is fully conserved in all Arabidopsis YUC homologs, and is essential for TR activity; however, the CXXC motif is not found in YUC6 (Cha et al., 2015). We therefore suggested that the TR activity of YUC6 would be involved in a catalytic mechanism that differs from those of other known NTRs (Cha et al., 2015). Our previous studies clearly showed that a mutation in YUC6-Cys85 abolished the YUC6–induced oxidative stress and drought tolerance, as well as delaying leaf senescence through the dysregulation of ROS homeostasis, without any changes in IAA biosynthesis (Cha et al., 2015; Cha et al., 2016). These biochemical and physiological phenomena were recapitulated in YUC6–mediated Ni stress tolerance. YUC6^C85S^-OX plants showed a loss of YUC6–induced Ni stress tolerance (**Figure 5**), suggesting that the Cys85–dependent TR activity of YUC6 is vital for acquiring Ni–stress tolerance. Furthermore, our previous finding revealed that the enhanced expression of *YUC6* in *yuc6-1D* and YUC6-OX plants elevated PRX activity, but not CAT activity, in the absence of stress conditions, suggesting that YUC6 may act upstream of *PRX* expression or the regulation of PRX activity (Cha et al., 2015). The current study also showed that the already enhanced PRX activity and *PrxQ* transcription in *yuc6-1D* were significantly further increased by Ni stress (**Figure 7**). YUC6 may therefore not only activate the transcription of *PrxQ* but also regulate the redox state of PRX through its TR activity, alleviating Ni stress–induced ROS accumulation.

**Figure 8:**
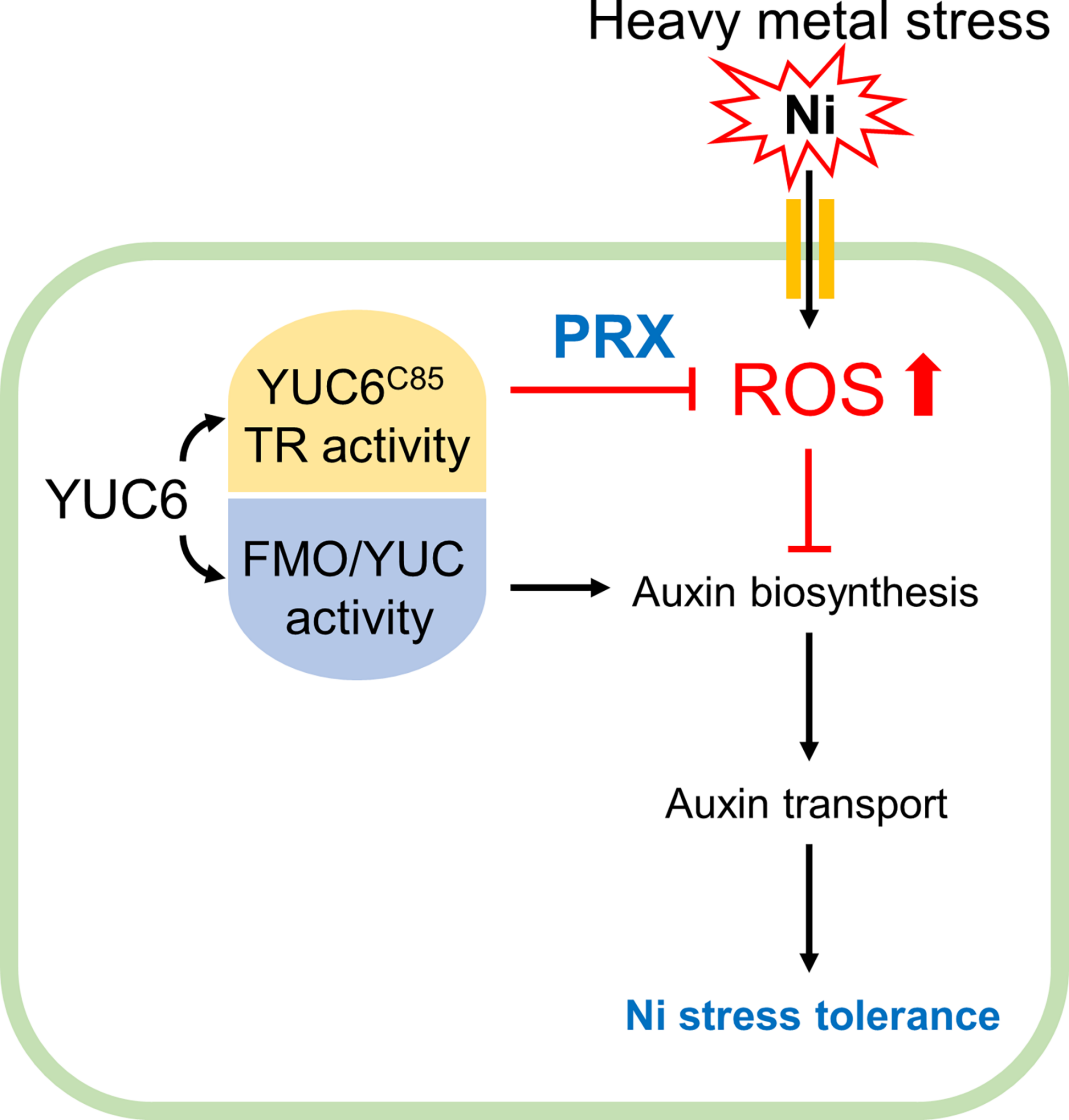
Proposed model for acquiring Ni stress tolerance through TR activity of YUC6 in Arabidopsis. Ni heavy metal stress mediates ROS production and inhibits auxin biosynthesis and distribution resulting in growth retardation. YUC6 has dual enzyme activities as an FMO/YUC activity and Cys85-dependent TR activity for auxin biosynthesis and ROS inhibition, respectively. Inhibition of auxin biosynthesis increased Ni toxicity, suggesting that maintenance of auxin homeostasis is necessary for basal Ni stress tolerance. Overexpression of YUC6 enhanced Ni stress tolerance in Arabidopsis, and its tolerance is due to the TR activity of YUC6. Thus, the TR activity of YUC6 is predominantly involved in Ni stress tolerance rather than elevated auxin levels in Arabidopsis. Furthermore, under Ni stress conditions, YUC6 promotes the antioxidant enzyme PRX activity and transcription of *PrxQ*. Collectively, YUC6 positively regulates Ni stress tolerance *via* its TR activity.

In conclusion, phytohormones are necessary for a basal tolerance to heavy metal stress; however, exogenous phytohormone (IAA, CK, and GA) applications can raise the antioxidant capacity, resulting in the reduction of heavy metal toxicity (Sytar et al., 2019). This is consistent with our data, which showed that the inhibition of IAA biosynthesis dramatically enhanced Ni stress sensitivity, while the exogenous application of IAA (concentration up to 1 µM) conferred Ni stress tolerance (**Figures 1**, **4**), suggesting that the maintenance of endogenous IAA levels through the auxin biosynthetic enzyme YUC is necessary for basal Ni stress tolerance (**Figure 8**). Moreover, the enhanced TR activity of YUC6 in the *yuc6-1D* and YUC6-OX plants conferred Ni stress tolerance *via* the activation of PRX specifically, rather than the elevated IAA levels, suggesting that the TR activity of YUC6 regulates Ni stress tolerance through the maintenance of ROS homeostasis (**Figures 5** **-** **8**). This mechanism by which YUC6 reduces Ni stress toxicity could be targeted in the development of plant materials for the elimination of toxic heavy metals from contaminated soils.

## Data availability statement

The original contributions presented in the study are included in the article/**Supplementary Material**. Further inquiries can be directed to the corresponding author.

## Author contributions

J-YC, SYJ, GA, SYL, D-JY, and W-YK designed the research; J-YC, SYJ, GA, G-IS, MGJ, SCL, DK, DMM, and YBL performed the experiments; J-YC, SYJ, GA, MGK, SYL, D-JY, and W-YK analyzed the data; J-YC, SYJ, GA, MGK, and W-YK wrote the paper. All authors contributed to the article and approved the submitted version.

## Funding

This research was supported by the National Research Foundation of Korea (NRF) grant funded by the Korean Government (MSIT-2022R1A5A1031361) and a Next-Generation BioGreen 21 Program (PJ01367101 to J-YC) funded by the Rural Development Administration (RDA), Republic of Korea.

## Acknowledgments

We thank Dr. Ho Byoung Chae for the kind gifts of recombinant Arabidopsis Trx-h2 and NTRA proteins and the BK21 four program for financial assistance.

## Conflict of interest

The authors declare that the research was conducted in the absence of any commercial or financial relationships that could be construed as a potential conflict of interest.

## Publisher’s note

All claims expressed in this article are solely those of the authors and do not necessarily represent those of their affiliated organizations, or those of the publisher, the editors and the reviewers. Any product that may be evaluated in this article, or claim that may be made by its manufacturer, is not guaranteed or endorsed by the publisher.
