# Supplementary material for: The thiol-reductase activity of YUCCA6 enhances nickel heavy metal stress tolerance in Arabidopsis
Source: Front Plant Sci. 2022 Sep 27;13:1007542. doi: 10.3389/fpls.2022.1007542 (PMC9551240; doi:10.3389/fpls.2022.1007542)
Supplement: Supplementary file 1 [file DataSheet_1.pdf]

Supplementary materials for

The thiol-reductase activity of YUCCA6 enhances nickel heavy metal stress tolerance in Arabidopsis

Joon-Yung Cha, Song Yi Jeong, Gyeongik Ahn, Gyeong-Im Shin, Myung Geun Ji, Sang Cheol Lee, Dhruba Khakurel, Donah Mary Macoy, Yong Bok Lee, Min Gab Kim, Sang Yeol Lee, Dae-Jin Yun, Woe-Yeon Kim

Correspondence to Woe-Yeon Kim
Email: kim1312@gnu.ac.kr

Supplementary materials contain two Supplementary tables and three Supplementary figures.

Supplementary Table 1. Primers used in this study.

Accession No.	Gene ID		Primer sequence
AT3G26060	(*PrxQ*)	Forward	5'- AGATGACTCTGCTTCTCACAAGGC
		Reverse	5'- TCCCTGGCAATGCTCCAAACAG
AT1G07890	(*APX1*)	Forward	5'- AGATGACTCTGCTTCTCACAAGGC
		Reverse	5'- TCCCTGGCAATGCTCCAAACAG
AT4G25100	(*FeSOD*, *FSD1*)	Forward	5'- GGATGTGTGGGAGCACTCTT
		Reverse	5'- GATTGGGATGTTGGGTTCAC
AT1G20630	(*CAT1*)	Forward	5'- GCCTGTCGGATGAGGAAGCTATCAGAG
		Reverse	5'- CCTAGGTTGGAACTTCCCTTCATTAAC
AT5G25620	(*YUC6*)	Forward	5'- GGGCTGTCCATGTTCTTACTGAA
		Reverse	5'- CGGGAAACAACCAAAAGGAA
AT1G08980	(*AMI1*)	Forward	5'-TTGGTTAAGGACACACTCGGCAG
		Reverse	5'-ACTCGATGATCCCCCAGGCACTC
AT3G44310	(*NIT1*)	Forward	5'-GGCGTTCATAACGAAGAAGGGCGTG
		Reverse	5'-TTCCTTCTCTATGGCTCCCATTACC
AT1G70560	(*TAA1*)	Forward	5'-AACGCTGCGACGGAGGATCG
		Reverse	5'-CGTGGACGGCGGCTTGACAA
AT4G39950	(*CYP79B2*)	Forward	5'-CCTCTTCTTCTCTATACACAAA
		Reverse	5'-GGTTTCTTTTTGTTGGGATCC
AT4G14560	(*IAA1*)	Forward	5'- CCTTTTGGTCGAGGCA
		Reverse	5'- TGTTAGTATCAAATATCTTGAGCAT
AT2G40220	(*ABI4*)	Forward	5'- GGGACAATTCCAACACCAAC
		Reverse	5'- TTACACCCACTTCCTCCTTG
AT3G20770	(*EIN3*)	Forward	5'- CCGATTGGACCGACTCCTCATAC
		Reverse	5'- ATAGCAAGCCAGGTAGCACTCTC
AT5G62690	(*TUBULIN2*)	Forward	5'- TGGCATCAACTTTCATTGGA
		Reverse	5'- ATGTTGCTCTCCGCTTCTGT
AT5G12240		Forward	5'- AGCGGCTGCTGAGAAGAAAGT
		Reverse	5'- TCTCGAAAGCCTTGCAAAATCT

Supplementary Table 2. ICP-MS analysis.

Cations	Treatment	Col-gl	*yuc6-1D*
K	Control	46.71 ± 0.06	47.34 ± 0.18
	Ni treatment	43.31 ± 0.52	44.21 ± 0.52
Ca	Control	5.75 ± 0.32	5.74 ± 0.13
	Ni treatment	5.54 ± 0.14	5.58 ± 0.02
Mg	Control	2.38 ± 0.02	2.42 ± 0.01
	Ni treatment	2.40 ± 0.02	2.38 ± 0.02
P	Control	6.71 ± 0.11	6.92 ± 0.11
	Ni treatment	6.65 ± 0.12	6.48 ± 0.08
Na	Control	0.74 ± 0.02	0.78 ± 0.01
	Ni treatment	0.76 ± 0.02	0.73 ± 0.08
Zn	Control	0.26 ± 0.01	0.30 ± 0.02
	Ni treatment	0.29 ± 0.02	0.26 ± 0.01
Mo	Control	9.23 ± 0.15	9.98 ± 0.37
	Ni treatment	9.71 ± 0.08	10.20 ± 0.06
Cu	Control	-0.48 ± 1.05	-0.55 ± 0.54
	Ni treatment	8.09 ± 2.40	8.49 ± 0.67
Cd	Control	0.31 ± 0.16	0.01 ± 0.04
	Ni treatment	-1.16 ± 0.06	-1.31 ± 0.08
Co	Control	0.28 ± 0.04	0.22 ± 0.12
	Ni treatment	0.46 ± 0.04	0.54 ± 0.11
Al	Control	29.76 ± 6.73	25.00 ± 1.64
	Ni treatment	27.01 ± 6.48	26.45 ± 0.86
Ba	Control	1.46 ± 0.36	1.72 ± 1.20
	Ni treatment	1.87 ± 1.31	1.88 ± 0.37
Pb	Control	2.89 ± 0.66	2.72 ± 0.32
	Ni treatment	4.35 ± 0.57	2.79 ± 0.46
Li	Control	22.63 ± 13.47	15.79 ± 1.27
	Ni treatment	13.01 ± 8.53	18.88 ± 4.37
Ti	Control	2.04 ± 1.04	2.00 ± 0.29
	Ni treatment	2.63 ± 0.88	2.97 ± 0.27
Si	Control	34.13 ± 33.09	-4.65 ± 2.95
	Ni treatment	5.12 ± 21.55	6.84 ± 16.51
Cr	Control	-24.23 ± 0.95	-11.34 ± 11.38
	Ni treatment	-21.10 ± 1.93	-22.20 ± 0.64
V	Control	-2.52 ± 0.08	-2.25 ± 0.10
	Ni treatment	-2.24 ± 0.16	-2.21 ± 0.08
B	Control	-11.56 ± 16.23	-2.40 ± 2.38
	Ni treatment	-7.11 ± 0.85	-3.18 ± 0.38

Supplementary Figure 1.

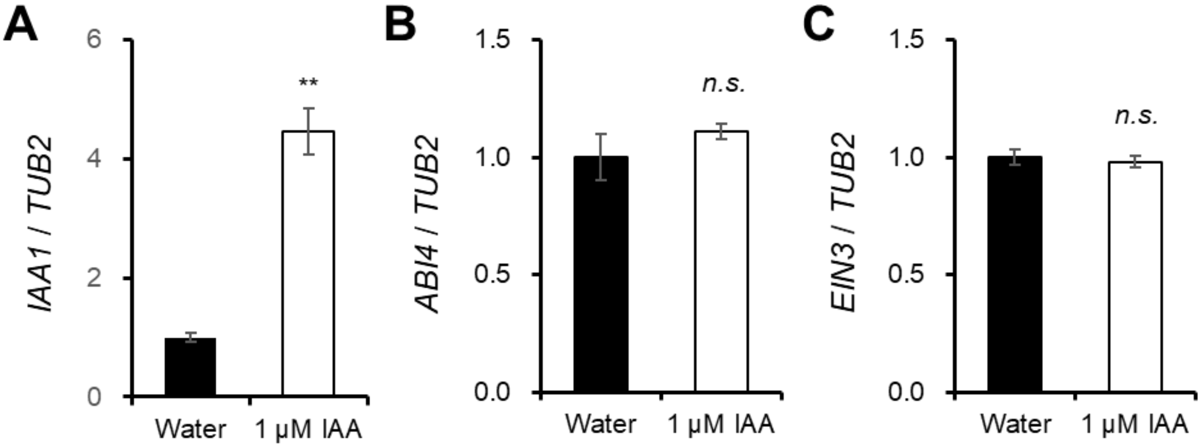

Supplementary Figure 1. Exogenous IAA treatments did not affect ABA and ETH.

Two-week-old Col-0 WT plants were treated with 1 μM IAA for 12 hrs. Expression levels of *IAA1* (to confirm the auxin treatment, A), *ABI4* (for ABA response, B), and *EIN3* (for ETH response, C) were measured by qPCR and normalized to *TUB2*. Values are means ± SE of four independent biological replicates. Asterisks indicate significant differences (** $p < 0.01$, and *n.s.*, no significant) from H_2O treatments determined by two-tailed Student's *t*-test.

Supplementary Figure 2.

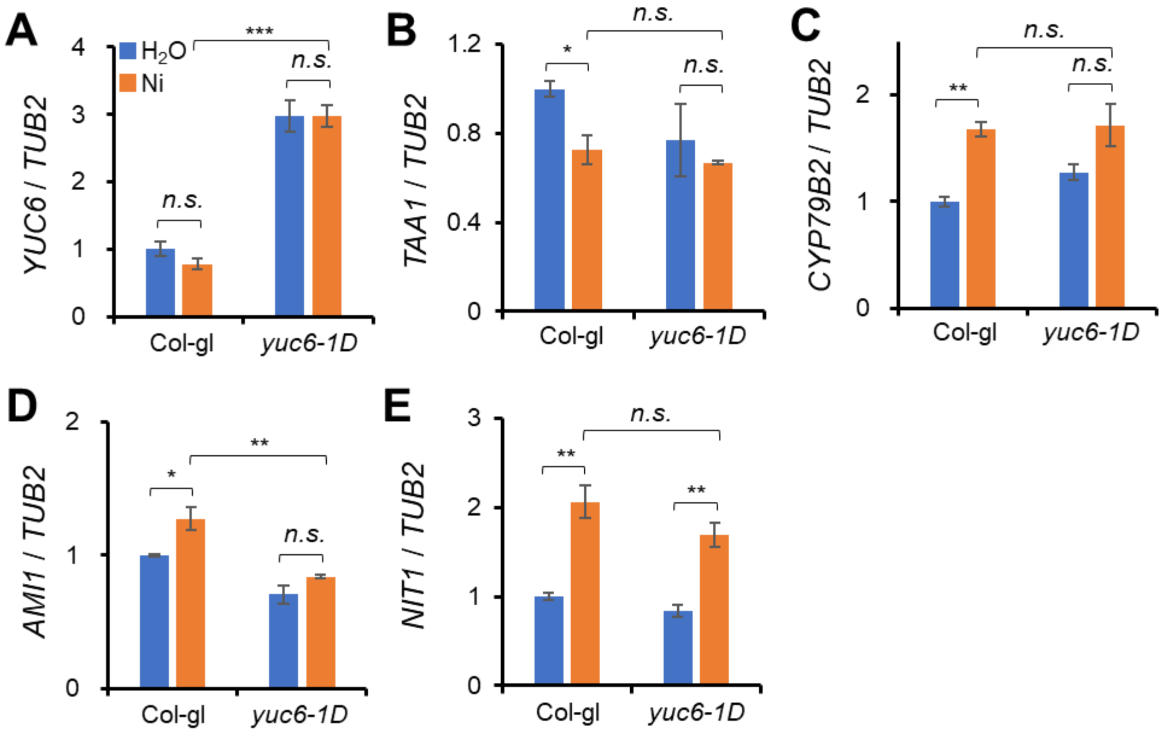

Supplementary Figure 2. YUC6-induced Ni stress tolerance is not due to the other auxin biosynthesis pathway.

Two-week-old Col-gl WT and *yuc6-1D* plants were treated with or without 100 µM Ni for 6 hrs. Expression levels of *YUC6* (to confirm the YUC6 overexpression, A), *TAA1* (for IPA pathway converting Trp to IPA, B), *CYP79B2* (for IAOx pathway converting Trp to IAOx, C), *AMI1* (for IAM pathway converting IAM to IAA, D), and *NIT1* (for IAN pathway converting IAN to IAA, E) were measured by qPCR and normalized to *TUB2*. Values are means ± SE of three independent biological replicates. Asterisks indicate significant differences (* $p < 0.05$, ** $p < 0.01$, *** $p < 0.001$, and *n.s.*, no significant) from H_2O treatments in each genotype or between Ni-treated Col-gl and *you6-1D* determined by two-tailed Student's *t*-test.

Supplementary Figure 3.

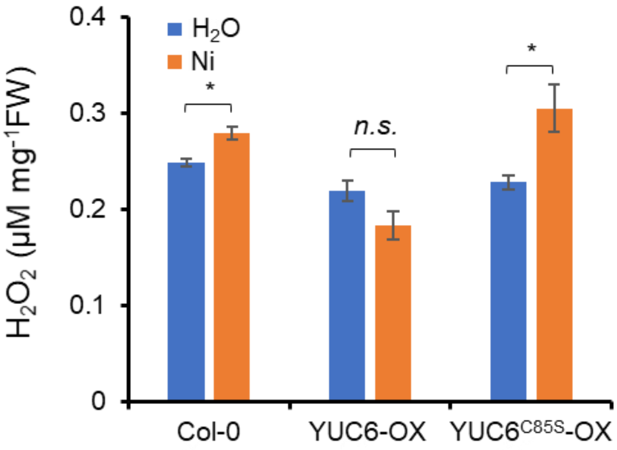

Supplementary Figure 3. TR activity of YUC6 is essential for the inhibition of Ni stress-induced ROS accumulation.

Two-week-old Col-0 WT, YUC6-OX, and YUC6^{C85S}-OX plants were treated with or without 100 μM Ni for 12 hrs and measured H_2O_2 contents. Values are means ± SE of three independent biological replicates. Asterisks indicate significant differences (* $p < 0.05$, and *n.s.*, no significant) from H_2O treatments in each plant determined by two-tailed Student's *t*-test.
